# A Universal Formulary

**Published:** 1854-11

**Authors:** 


**A UNIVERSAL FORMULARY—Containing the methods of preparing and** administering officinal and other medicines. The whole adapted to Physicians and Pharmaceutists by R. EGGLESFIELD GRIFFITH, M. D. A NEW edition carefully revised and much extended by ROBERT P. THOMAS, M. D. With illustrations. "Selecta sunt quæ Medicum Nobilitant."—Linneas. Philadelphia: Blanchard & Lea, 1854. pp. 651.

We of the present day are greatly amused by the disgusting and interminable messes prescribed by the physicians of former years for the cure of diseases; but even in this enlightened age there are forced down the throats of patients many mixtures quite as incongruous and as inert as those which now excite our laughter.

This arises from the neglect, by physicians, of the art of prescribing—a neglect which we are satisfied is a fruitful cause of ill success in practice. Of course no formulary will enable the physician to determine whether the case before him demands a purgative, a narcotic, or a diaphoretic, *that* he must determine for himself; but when he has determined it, a careful study of any of the different authors who have written of late upon the subject, will enable him to apply his remedy in the most efficient form, avoiding cumbrousness, unpleasantness of taste as much as possible, and above all, avoiding incompatibility.

The work before us is, we think, admirably fitted to fulfil the purpose for which it is designed. It opens with an introduction containing full tables of the weights and measures of France, England, and the United States, and their proportions and relations to each other, together with a great deal of valuable information upon the specific gravities of different substances, a glossary of Latin terms used in prescribing, and sundry matters of interest to the physician and pharmaceutist. Following this is the main body of the work containing a vast number of valuable formulæ among which the physician may find the most approved form of giving any medicine he wishes to use. The work contains a most valuable index both of the formulæ and the diseases for which they have been advised.

